# Evaluation of Outcomes Between the Top-down Versus the Bottom-up Approach for Retropubic Midurethral Sling

**DOI:** 10.1007/s00192-024-05731-5

**Published:** 2024-02-20

**Authors:** Vidushri Mehrotra, John Pearl, David Sheyn, Susan D. Wherley

**Affiliations:** 1 Case Western Reserve University School of Medicine, Cleveland, OH USA; 2 Female Pelvic Medicine, Urology Institute, University Hospitals Cleveland Medical Center, Cleveland, OH USA; 3 Urogynecology and Reconstructive Pelvic Surgery Fellow, Urology Institute, University Hospitals Cleveland Medical Center, Cleveland, OH USA

**Keywords:** Midurethral sling, Retropubic sling, Surgical outcomes, Stress urinary incontinence

## Abstract

**Introduction and hypothesis:**

Retropubic midurethral sling (MUS) placement is the gold standard for the treatment of stress urinary incontinence in the USA. The procedure can be approached from either a top-down or a bottom-up direction, but there is a paucity of contemporary data regarding outcomes between these approaches. The aim of this study was to provide updated clinical outcomes data.

**Methods:**

This was a retrospective cohort study of women undergoing the retropubic MUS procedure alone or at the time of pelvic organ prolapse repair between 2010 and 2020 at a single academic medical center. The electronic medical record was used to extract demographic data, operative approach, and perioperative complications. The primary outcome was a composite incidence of any perioperative complication.

**Results:**

Of the 309 patients analyzed, 140 (45.3%) underwent top-down and 169 (54.7%) underwent bottom-up retropubic MUS placement. Patients undergoing top-down MUS placement were more likely to be older (mean age 58 vs 54, *p*=0.02), have a history of diabetes mellitus (20% vs 8.9%, *p*=0.004), and have had a prior hysterectomy (27% vs 16%, *p*=0.02). They were less likely to have a concurrent anterior (*p*<0.001) or posterior repair (*p*<0.001). Patients undergoing the top-down procedure were less likely to experience sling exposure (*p*=0.02); complications in the two groups were otherwise similar.

**Conclusions:**

The top-down approach to retropubic MUS placement was associated with lower rates of mesh erosion in this population of patients. Neither approach is associated with an increased overall risk of complications or de novo overactive bladder symptoms.

## Introduction

Recent studies estimate that nearly half of adult women experience stress urinary incontinence (SUI), with 13.6% undergoing surgical treatment during their lifetime [1, 2]. The incidence of patients undergoing primary SUI surgery is expected to rise with increasing patient awareness and education of treatment options for this common condition, which has detrimental lifestyle effects. Women diagnosed with stress incontinence tend to avoid social situations, limit their physical activity, have worse mental health, and have decreased sexual function, all of which contribute to a decreased quality of life [3–5]. The gold standard primary treatment option for women diagnosed with bothersome stress urinary incontinence in the USA is synthetic midurethral sling (MUS) placement. Although each type of MUS has benefits and risks, retropubic slings have the highest rates of both short-term and long-term efficacy and are used most commonly [6, 7]. The retropubic MUS procedure can be approached from either a top-down or a bottom-up direction, based on the initial trocar placement. In the top-down procedure, two needle trocars are inserted through small abdominal incisions and passed through the retropubic space, exiting through the midline vaginal incision. The bottom-up procedure is the opposite, wherein the two needle trocars are inserted through the midline vaginal incision and passed through the retropubic space, exiting through the abdominal wall. These two approaches have historically been treated as equivalent, numerous systems exist in the marketplace for each, and the decision regarding approach has been a matter of surgeon preference.

A series of small randomized controlled trials published from 2004 to 2006 sought to determine whether complications with retropubic MUS placement were attributable to either the top-down or the bottom-up approach. These five trials, including 636 patients, looked specifically at outcomes between the top-down Suprapubic Arch Sling System (American Medical Systems, Minnetonka, MN, USA) and the bottom-up Tension-free Vaginal Tape (Johnson and Johnson, New Brunswick, NJ, USA). A Cochrane Database meta-analysis found that the top-down approach was associated with increased rates of voiding dysfunction, bladder perforation, and vaginal mesh exposures [8]. However, no additional trials have been conducted since the early 2000s, despite the training of hundreds of additional urogynecology subspecialists, the refinement of training and operative technique, and the appearance of more than a dozen new sling systems on the market. Prior trials also excluded procedures where concomitant prolapse repair was performed.

The aim of this study, therefore, was to provide updated clinical data to guide future treatment and training. Our first objective was to compare short-term and long-term outcomes for these two approaches. Our second objective was to explore whether sling approach was associated with de novo symptomatic overactive bladder (OAB) and subsequent treatment of this condition.

## Materials and Methods

This was a retrospective cohort study of women undergoing retropubic MUS procedure alone or at the time of pelvic organ prolapse (POP) repair between 1 January 2010 and 31 December 2020 at a single academic medical center with three fellowship-trained urogynecology surgeons. This study was approved by the Institutional Review Board at the University Hospitals Cleveland Medical Center. Patients were identified for inclusion using the Current Procedural Terminology code for MUS placement, 58278. A detailed review of the operative report was performed and patients undergoing concomitant oncological or nongynecological procedures, transobturator MUS placement, or single-incision MUS placement were excluded. Patients who had incomplete data regarding the type of sling or surgical approach and patients with a history of neurogenic bladder were similarly excluded. A total of 438 patients who underwent MUS placement were identified and after records were reviewed, 309 patients were found to meet all study criteria and have complete records. Patients who had a positive cough stress test in the office or demonstrated stress urinary incontinence on urodynamic testing were considered candidates for sling placement. Concomitant POP repair was performed in patients with symptomatic POP. Surgical approach was determined by individual surgeon preference, with one surgeon with a urology background preferring the top-down approach and two surgeons with a gynecology background preferring the bottom-up approach.

For each patient undergoing MUS placement, the electronic medical record was used to extract demographic data, operative approach, and perioperative complications. The primary outcome was a composite incidence of any perioperative complication, including postoperative urinary tract infection or pelvic hematoma within 90 days of surgery, failure of trial of void, sling exposure, sling lysis or need for repeat sling placement. Secondary outcomes included the incidence of de novo OAB symptoms defined as de novo urinary urgency with or without frequency, nocturia, and urgency incontinence occurring more than 90 days but before 1 year following the primary procedure and incidence of OAB requiring treatment with behavioral modification, pelvic floor therapy, medication, percutaneous tibial nerve stimulation, intradetrusor OnabotulinumtoxinA injection, or sacral neuromodulation. Additional demographic variables included in the analysis were: age, body mass index, mode of delivery, prior pelvic surgery, diuretic use, diabetes, preoperative OAB symptoms, presence of detrusor overactivity on preoperative urodynamic testing, and concomitant procedures for hysterectomy or pelvic organ prolapse repair.

An a priori power analysis determined that we would need 140 patients in each group to identify a 10% difference in complication rates with 80% power and an alpha-error of 5%. Descriptive statistics were expressed as median and interquartile range or mean and standard deviation where appropriate. Group comparison was performed using Wilcoxon rank-sum, Student’s *t* test, and Fisher’s exact test as appropriate. Multivariate logistic regression was used to evaluate for independent predictors of sling-related complications and for de novo OAB. Variables with a *p* value of < 0.20 on univariate analysis were included in the multivariate model. A *p* value of <0.05 was considered to be statistically significant. Statistical analysis was performed using STATA version 14.1 (Stata Corp, College Station, TX, USA).

## Results

Of the 309 women who underwent retropubic MUS placement between 2010 and 2020, a total of 140 (45.3%) underwent a top-down procedure and 169 (54.7%) underwent a bottom-up procedure. Table 1 displays patient demographics. Patients undergoing top-down MUS placement were more likely to be older (mean age 58 vs. 54, *p*=0.02), have a history of diabetes mellitus (20% vs 8.9%, *p*=0.004), and have had a prior hysterectomy (27% vs 16%, *p*=0.02) than patients undergoing bottom-up MUS placement. Other demographic factors such as mode of prior deliveries, smoking status, menopausal status, prior prolapse repair, prior sling, diuretic use, preoperative overactive bladder symptoms, and detrusor overactivity on preoperative urodynamic testing were similar between the two surgical groups.

**Table 1:** Patient demographics

	Top-down, *N*=140 (%)	Bottom-up, *N*=169 (%)	*p* value
Age, median (IQR)	58 (48–68)	54 (47–65)	0.02
Body mass index	26.5	27.8	0.54
Mode of delivery	–	–	0.83
Cesarean only	10 (7.1)	7 (4.1)	–
Vaginal only	97 (69.2)	119 (70.4)	–
Both vaginal and cesarean delivery	33 (23.6)	43 (25.4)	–
Current smoker	20 (14.3)	15 (8.9)	0.14
Post-menopausal	98 (70)	101 (59.8)	0.06
Systemic hormone use	11 (7.9)	21 (12.4)	0.13
Vaginal estrogen use	34 (24.2)	37 (21.9)	0.26
Prior prolapse repair	0	3 (1.8)	0.16
Prior sling	–	–	0.29
Retropubic synthetic sling	10 (7.1)	6 (3.6)	–
Pubovaginal sling	0	1 (0.6)	–
Mini-sling	0	1 (0.6)	–
Prior hysterectomy	38 (27)	27 (16)	0.02
Diuretic use	22 (15.7)	36 (21.3)	0.67
Diabetes mellitus	28 (20)	15 (8.9)	0.004
Preoperative overactive bladder symptoms	61 (43.6)	61 (36)	0.43
Detrusor overactivity on preoperative urodynamic testing	10 (7.1)	11 (6.5)	0.69

*IQR* interquartile range

Table 2 lists concomitant procedures performed at the time of MUS placement. Patients who underwent top-down MUS placement were less likely to have a concurrent anterior (*p*<0.001) or posterior repair (*p*<0.001) compared with patients who had the bottom-up procedure. There were no significant differences found in rates of concurrent sacrocolpopexy, uterosacral suspension, obliterative procedure, salpingectomy, and hysterectomy.

**Table 2:** Concomitant procedures

	Top-down, *N*=140 (%)	Bottom-up, *N*=169 (%)	*p* value
ASA class, median (IQR)	2 (1–3)	2 (1–3)	0.13
Anterior repair	13 (9.3)	79 (46.7)	<0.001
Posterior repair	22 (15.7)	83 (49.1)	<0.001
Sacrocolpopexy	17 (12.1)	31 (18.3)	0.13
Uterosacral suspension	13 (9.3)	13 (7.7)	0.38
Obliterative procedure	6 (4.3)	4 (2.4)	0.27
Salpingectomy	16 (11.4)	26 (15.4)	0.43
Concurrent hysterectomy	28 (20)	39 (23.1)	0.51

*ASA* American Society of Anesthesiologists, *IQR* interquartile range

Table 3 shows perioperative complications. Complications in the two groups were similar, with the exception of sling exposures. Patients undergoing the top-down procedure were less likely to experience sling exposure (*p*=0.02), and all top-down exposures were classified as International Urogynecological Association (IUGA) category 2B (symptomatic vaginal exposures ≥1 cm). Exposures associated with the bottom-up procedure included IUGA category 2A, 2B, and 3B complications. Short-term surgical outcomes and longer-term urinary symptoms in the two groups were also similar.

**Table 3:** Procedure outcomes

	Top-down, *N*=140 (%)	Bottom-up, *N*=169 (%)	*p* value
UTI within 90 days	19 (13.6)	28 (16.6)	0.5
Blood transfusion	3 (2.1)	0 (0.0)	0.09
Pelvic hematoma	2 (1.4)	2 (1.2)	0.61
Bladder perforation	5 (3.6)	6 (3.6)	0.6
Failed trial of void	21 (15)	19 (11.2)	0.18
Sling erosion	2 (1.4)	11 (6.5)	0.02
Sling lysis due to obstruction	1 (0.7)	2 (1.2)	0.57
Required second sling	2 (1.4)	2 (1.2)	0.588
Any de novo OAB symptoms	19 (13.6)	27 (16)	0.54
De novo nocturia	6 (4.3)	7 (4.1)	0.06
De novo urgency	11 (7.9)	11 (6.5)	0.54
De novo frequency	8 (5.7)	14 (8.3)	0.96
De novo urge incontinence	8 (5.7)	15 (8.9)	0.89
OAB symptoms resolved spontaneously	6 (4.3)	7 (4.1)	0.49
UDS performed postoperatively	17 (12.1)	20 (11.8)	0.49
De novo detrusor overactivity on urodynamic testing	2 (1.4)	8 (4.7)	0.14
OAB treated with medication	22 (15.7)	23 (13.6)	0.63
OAB treated with physical therapy	9 (6.4)	12 (7.1)	0.51
OAB treated with third-line therapy	3 (2.1)	6 (3.6)	0.31

*UTI* urinary tract infection, *OAB* overactive bladder, *UDS* urodynamic studies

Table 4 demonstrates the regression analysis for independent predictors of sling-related complications and for de novo OAB. Concurrent anterior repair was associated with a higher risk of sling-related complications (aOR=1.43, 95% CI: 1.16–1.97). Neither approach to retropubic MUS placement was associated with a higher complication rate (aOR=1.55, 95% CI: 0.71–3.38). For the secondary outcome of de novo OAB, vaginal delivery was associated with a decreased risk of OAB compared with cesarean delivery (aOR=0.31, 95% CI: 0.21–0.89), and being post-menopausal was associated with a higher risk of de novo OAB (aOR=2.32, 95% CI: 1.07–5.36).

**Table 4:** Regression analysis for risk of complications of midurethral sling (*MUS*)

	Unadjusted OR (95% CI)	Adjusted OR (95% CI)
Age per every 10 years	1.13 (0.98–1.37)	1.03 (0.73–1.43)
Mode of delivery	0.90 (0.54–1.51)	–
Current smoker	0.60 (0.26–1.39)	0.68 (0.17–2.65)
Post-menopausal	1.03 (0.62–1.71)	–
Prior prolapse repair	1.07 (0.09–12.03)	–
Prior sling	3.89 (1.36–11.01)	4.01 (0.72–22.27)
Prior hysterectomy	1.23 (0.69–2.19)	–
Diuretic use	0.51 (0.25–1.07)	0.54 (0.21–1.41)
Diabetes mellitus	1.21 (0.61–2.38)	0.51 (0.13–1.93)
Preoperative overactive bladder symptoms	1.05 (0.64–1.73)	–
Detrusor overactivity on preoperative urodynamic testing	0.63 (0.24–1.83)	–
ASA class, median (IQR)	1.05 (0.67–1.63)	–
Anterior repair	1.68 (1.17–1.39)	1.43 (1.16–1.97)
Posterior repair	1.05 (0.63–1.73)	–
Sacrocolpopexy	1.09 (0.57–2.10)	–
Uterosacral suspension	1.38 (0.60–3.17)	–
Obliterative procedure	2.21 (0.63–7.83)	–
Salpingectomy	1.01 (0.49–2.06)	–
Concurrent hysterectomy	1.26 (0.71–2.23)	–
Top-down approach	1.10 (0.68–1.78)	1.55 (0.71–3.38)

*ASA* American Society of Anesthesiologists, *IQR* interquartile range

## Discussion

The aim of this retrospective cohort study was to explore whether patient outcomes differ depending on surgical approach to midurethral sling placement. Regarding our primary outcome of perioperative complications, we found that the only significant difference between the surgical approaches was a greater incidence of sling erosion and mesh exposure with the bottom-up approach. These vaginal exposures were also more likely to be greater than 1 cm. This contrasts with prior studies, which have shown higher rates of mesh exposure with a top-down approach [8–10]. This difference may be attributable to surgeon experience or technique, but prior studies have not suggested theories for their findings. Given that the top-down approach generally requires a larger dissection to allow a gloved finger to reach the pubic ramus, this dissection may result in improved tissue mobility and facilitate a tension-free closure that results in a lower rate of mesh exposure. However, the prior studies comparing complications of the two approaches included cases where only sling insertion was performed. The fact that in this study’s cohort nearly half of patients who underwent bottom-up sling placement had concurrent prolapse surgery likely contributes to the higher rate of mesh exposure. This is consistent with the regression data, which confirm an increased risk of complications with anterior repair. Indeed, the higher rate of mesh exposure noted with the bottom-up approach is more likely related to the higher rates of anterior repair in this group. Although different incisions are created for anterior repair and midurethral sling placement—and surgeons who perform these procedures concomitantly ensure separation of at least 1 cm between incisions—it is plausible that the presence of an additional incision on the anterior wall of the vagina could lead to an increased risk of mesh exposure in the future. This finding suggests that surgeons might consider avoiding MUS placement with concomitant anterior repair and instead plan for staged procedures. While multiple studies have evaluated immediate postoperative complications in patients undergoing concomitant prolapse repair and MUS placement, the majority of these studies have endeavored to answer the question of whether prophylactic slings or native tissue repair are efficacious for treatment of possible occult stress incontinence, as these anti-incontinence procedures have known risks of complication [11]. Most report similar perioperative complication rates overall, although they were not powered for detecting differences in complications and did not report on long-term outcomes including mesh exposures [12–14]. van der Ploeg et al. found that patients who underwent concomitant sling and prolapse procedures had a higher rate of mesh exposure, but did not stratify outcomes based on specific prolapse procedures. However, given that the majority of patients (between 86% and 92%) underwent anterior repair at the time of MUS placement, this data may corroborate our findings [15, 16].

There were no other significant differences in the incidence of other complications and outcomes between the top-down and bottom-up surgical approaches, including urinary tract infection, peri-operative blood transfusion, pelvic hematoma, failed trial of void, therapeutic sling lysis, and second sling procedure. This also differs from the prior Cochrane meta-analysis, which found higher rates of voiding dysfunction and bladder perforation in the top-down approach. Analysis of our secondary outcome of de novo OAB post-MUS found no difference between the two surgical approaches. Of note, there was no difference in pre-operative OAB symptoms between the two patient groups. Further analysis of de novo OAB in the cohort found that after controlling for major risk factors, vaginal deliveries were protective against de novo OAB symptoms and post-menopausal status is associated with a higher risk of de novo OAB. This is consistent with studies that have shown that cesarean delivery is associated with OAB later in life [17, 18]. Vaginal delivery may be protective owing to avoidance of nerve damage associated with operative delivery or cesarean delivery. Another theory is that the tissue stress and remodeling associated with vaginal delivery and recovery could result in strengthening of the pelvic floor musculature. The decrease in systemic estrogen associated with the menopausal transition results in decreased elasticity and increased tissue atrophy. These effects on the musculature and connective tissue of the bladder and urethra contribute to higher rates of OAB in post-menopausal patients. Conversely, the use of systemic hormone replacement therapy or vaginal estrogen has been demonstrated to be protective against symptoms of post-menopausal OAB [19].

Although this is the largest study to date comparing the different surgical approaches to retropubic MUS placement, the inclusion of patients undergoing concomitant prolapse repair is both a strength and a limitation of this study. Although concomitant prolapse repair is frequently performed in patients undergoing MUS placement and it is essential to research outcomes for these patients, prolapse repair also serves as a confounding variable, making it difficult to ascertain whether the differences between approaches are related to the approach or to the concomitant prolapse repair. Other strengths of this study include the fact that it was sufficiently powered to detect differences in outcomes between the two approaches, the wide range of outcomes considered in the analysis, and the inclusion of only fellowship-trained urogynecologists at an academic medical center. We acknowledge that the study has several additional limitations, including bias inherent to the retrospective design and the impact of individual surgeon practice patterns on the results.

In conclusion, the top-down approach to retropubic MUS placement was associated with lower rates of mesh exposure in this population of patients undergoing MUS placement with or without concomitant prolapse repair. This result conflicts with the Cochrane meta-analysis of studies from the early 2000s, which found that the bottom-up method was associated with decreased complication rates, including mesh exposure [8]. Our study’s findings otherwise affirm that both techniques of retropubic MUS placement are associated with similarly low complication rates, further supporting the clinical recommendation that surgeons proceed via the approach that is more familiar to them. Future studies evaluating the surgical outcomes of the top-down and bottom-up approaches should consider a prospective design and follow patients for an extended period in order to evaluate longer-term outcomes of the two procedures. Future studies may also include new variables, such as operative time and cost, given the similar rates of postoperative complications between groups and the importance of a systems-based approach to clinical practice.

## References

[1] Abufaraj M, Xu T, Cao C (2021). Prevalence and trends in urinary incontinence among women in the United States, 2005–2018. Am J Obstet Gynecol.

[2] Wu JM, Matthews CA, Conover MM, Pate V, Jonsson Funk M (2014). Lifetime risk of stress urinary incontinence or pelvic organ prolapse surgery. Obstet Gynecol.

[3] Corrado B, Giardulli B, Polito F, Aprea S, Lanzano M, Dodaro C (2020). The impact of urinary incontinence on quality of life: a cross-sectional study in the Metropolitan City of Naples. Geriatrics (Basel).

[4] Mallah F, Montazeri A, Ghanbari Z, Tavoli A, Haghollahi F, Aziminekoo E (2014). Effect of urinary incontinence on quality of life among Iranian women. J Family Reprod Health.

[5] Naumann G, Hitschold T, Frohnmeyer D, Majinge P, Lange R (2021). Sexual disorders in women with overactive bladder and urinary stress incontinence compared to controls: a prospective study. Geburtshilfe Frauenheilkd.

[6] Richter HE, Albo ME, Zyczynski HM (2010). Retropubic versus transobturator midurethral slings for stress incontinence. N Engl J Med.

[7] Imamura M, Hudson J, Wallace SA (2019). Surgical interventions for women with stress urinary incontinence: systematic review and network meta-analysis of randomised controlled trials. BMJ.

[8] Ford AA, Rogerson L, Cody JD, Ogah J (2017). Mid-urethral sling operations for stress urinary incontinence in women. Cochrane Database Syst Rev.

[9] Andonian S, Chen T, St-Denis B, Corcos J (2005). Randomized clinical trial comparing suprapubic arch sling (SPARC) and tension-free vaginal tape (TVT): one-year results. Eur Urol.

[10] Lord HE, Taylor JD, Finn JC (2006). A randomized controlled equivalence trial of short-term complications and efficacy of tension-free vaginal tape and suprapubic urethral support sling for treating stress incontinence. BJU Int.

[11] Wei JT, Nygaard I, Richter HE, Nager CW, Barber MD, Kenton K (2012). A midurethral sling to reduce incontinence after vaginal prolapse repair. N Engl J Med..

[12] Meschia M, Pifarotti P, Spennacchio M, Buonaguidi A, Gattei U, Somigliana E (2004). A randomized comparison of tension-free vaginal tape and endopelvic fascia plication in women with genital prolapse and occult stress urinary incontinence. Am J Obstet Gynecol..

[13] Togami JM, Chow D, Winters JC (2010). To sling or not to sling at the time of anterior vaginal compartment repair. Curr Opin Urol..

[14] Schierlitz L, Dwyer PL, Rosamilia A, De Souza A, Murray C, Thomas E, et al. Pelvic organ prolapse surgery with and without tension-free vaginal tape in women with occult or asymptomatic urodynamic stress incontinence: a randomised controlled trial. Int Urogynecol J. 2014;25(1):33–40. 10.1007/s00192-013-2150-7. 10.1007/s00192-013-2150-7 23812579

[15] van der Ploeg JM, Oude Rengerink K, van der Steen A, van Leeuwen JH, Stekelenburg J, Bongers MY (2015). Transvaginal prolapse repair with or without the addition of a midurethral sling in women with genital prolapse and stress urinary incontinence: a randomised trial. BJOG..

[16] van der Ploeg JM, Oude Rengerink K, van der Steen A, van Leeuwen JH, van der Vaart CH, Roovers JP (2016). Vaginal prolapse repair with or without a midurethral sling in women with genital prolapse and occult stress urinary incontinence: a randomized trial. Int Urogynecol J..

[17] Gomelsky A, Steckenrider H, Dmochowski RR (2022). Urgency and urgency incontinence following stress urinary incontinence surgery: a review of evaluation and management. Indian J Urol.

[18] Marcelissen T, Van Kerrebroeck P (2018). Overactive bladder symptoms after midurethral sling surgery in women: risk factors and management. Neurourol Urodyn.

[19] Tomaszewski J (2014). Postmenopausal overactive bladder. Prz Menopauzalny.

